# Opfi: A Python package for identifying gene clusters in large genomics and metagenomics data sets

**DOI:** 10.21105/joss.03678

**Published:** 2021-10-27

**Authors:** Alexis M. Hill, James R. Rybarski, Kuang Hu, Ilya J. Finkelstein, Claus O. Wilke

**Affiliations:** 1 Department of Integrative Biology, The University of Texas at Austin, Austin, Texas 78712, USA; 2 Department of Molecular Biosciences, The University of Texas at Austin, Austin, Texas 78712, USA; 3 Center for Systems and Synthetic Biology, The University of Texas at Austin, Austin, Texas, 78712, USA

## Abstract

Gene clusters are sets of co-localized, often contiguous genes that together perform specific functions, many of which are relevant to biotechnology. There is a need for software tools that can extract candidate gene clusters from vast amounts of available genomic data. Therefore, we developed Opfi: a modular pipeline for identification of arbitrary gene clusters in assembled genomic or metagenomic sequences. Opfi contains functions for annotation, de-deduplication, and visualization of putative gene clusters. It utilizes a customizable rule-based filtering approach for selection of candidate systems that adhere to user-defined criteria. Opfi is implemented in Python, and is available on the Python Package Index and on Bioconda (Grüning et al., 2018).

## Statement of need

Gene clusters have been successfully repurposed for a number of biotechnical applications, including biofuel production, organic compound synthesis, and gene editing (Fischbach & Voigt, 2010). Despite the broad utility of known gene clusters, identification of novel gene clusters remains a challenging task. While there are many tools available for annotation of singular genes (or protein domains) in biological sequence data (Buchfink et al., 2021; Camacho et al., 2009; Steinegger & Söding, 2017), these programs do not identify whole gene clusters out of the box. In many cases, researchers must combine bioinformatics tools ad hoc, resulting in one-off pipelines that can be difficult to reproduce. Several software packages have been developed for the discovery of specific types of gene clusters (Blin et al., 2019; Santos-Aberturas et al., 2019; van Heel et al., 2018), but these tools may not be sufficiently flexible to identify clusters of an arbitrary genomic composition. To address these gaps, we developed a modular pipeline that integrates multiple bioinformatics tools, providing a flexible, uniform computational framework for identification of arbitrary gene clusters. In a recent study, we used Opfi to uncover novel CRISPR-associated transposons (CASTs) in a large metagenomics dataset (Rybarski et al., 2021).

## Implementation

Opfi is implemented in Python, and uses several bioinformatics tools for feature annotation (Buchfink et al., 2021; Camacho et al., 2009; Edgar, 2007; Shi & Liang, 2019; Steinegger & Söding, 2017). Users can install Opfi and all of its dependencies through Bioconda (Grüning et al., 2018). Opfi consists of two major components: Gene Finder, for discovery of gene clusters, and Operon Analyzer, for rule-based filtering, deduplication, and visualization of gene clusters identified by Gene Finder. All modules generate output in a comma-separated (CSV) format that is common to the entire package.

### Example Gene Finder usage

The following example script searches for putative CRISPR-Cas loci in the genome of *Rippkaea orientalis PCC 8802*. Information about the biological significance of this example, as well as data inputs and descriptions, can be found in the tutorials directory in the project GitHub repository. The example illustrates the use of the Pipeline class for setting up a gene cluster search. First, add_seed_step specifies a step to annotate *cas1* genes, using protein BLAST (BLASTP) (Camacho et al., 2009) and a database of representative Cas1 protein sequences. 10,000 bp regions directly up- and downstream of each putative *cas1* gene are selected for further analysis, and all other regions are discarded. Next, add_filter_step adds a step to annotate candidate regions for additonal *cas* genes. Candidates that do not have at least one additional *cas* gene are discarded from the master list of putative systems. Finally, add_crispr_step adds a step to search remaining candidates for CRISPR arrays, i.e. regions of alternating ~30 bp direct repeat and variable sequences, using the PILER-CR repeat finding software (Edgar, 2007).


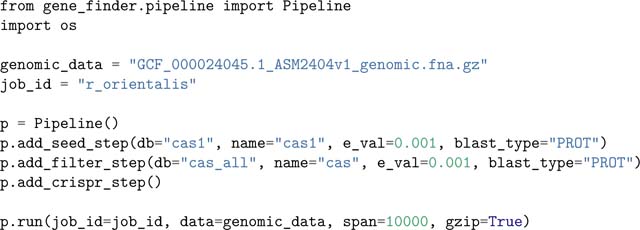


Running this code creates the CSV file r_orientalis_results.csv, which contains information about each system identified; in this example, that is two canonical CRISPR-Cas systems, and one locus with weak homology to *cas* genes. Each line in the file represents a single putative feature in a candidate locus. Features from the same candidate are grouped together in the CSV. Detailed information about the output format can be found in the Opfi documentation.

### Example Operon Analyzer usage

In the previous example, passing systems must meet the relatively permissive criterion of having at least one *cas1* gene co-localized with one additional *cas* gene. This is sufficient to identify CRISPR-Cas loci, but may also capture regions that do not contain functional CRISPR-Cas systems, but rather consist of open reading frames (ORFs) with weak homology to *cas* genes.

These improbable systems could be eliminated during the homology search by making the match acceptance threshold more restrictive (i.e., by decreasing the e-value), however, this could result in the loss of interesting, highly diverged systems. Therefore, we implemented a module that enables post-homology search filtering of candidate systems, using flexible rules that can be combined to create sophisticated elimination functions. This allows the user to first perform a broad homology search with permissive parameters, and then apply rules to cull unlikely candidates without losing interesting and/or novel systems. Additionally, rules may be useful for selecting candidates with a specific genomic composition for downstream analysis. It should be noted that the use of the term “operon” throughout this library is an artifact from early development of Opfi. At this time, Opfi does not predict whether a candidate system represents a true operon, that is, a set of genes under the control of a single promoter. Although a candidate gene cluster may certainly qualify as an operon, it is currently up to the user to make that distinction.

Rule-based filtering is illustrated with the following example. The sample script takes the output generated by the previous example and reconstructs each system as an Operon object. Next, the RuleSet class is used to assess each candidate; here, passing systems must contain two cascade genes (*cas5* and *cas7*) no more than 1000 bp apart, and at least one *cas3* (effector) gene. For a complete list of rules, see the Opfi documentation.


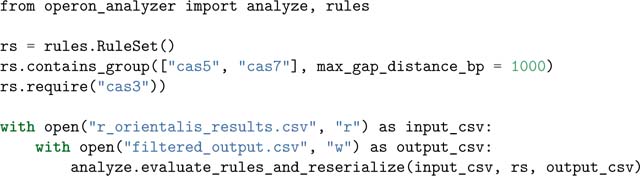


After running this code, the file filtered_output.csv contains only high-confidence type-I CRISPR-Cas systems (re-serialized to CSV format) that passed all rules in the rule set.

### Candidate visualization

**Figure 1:**
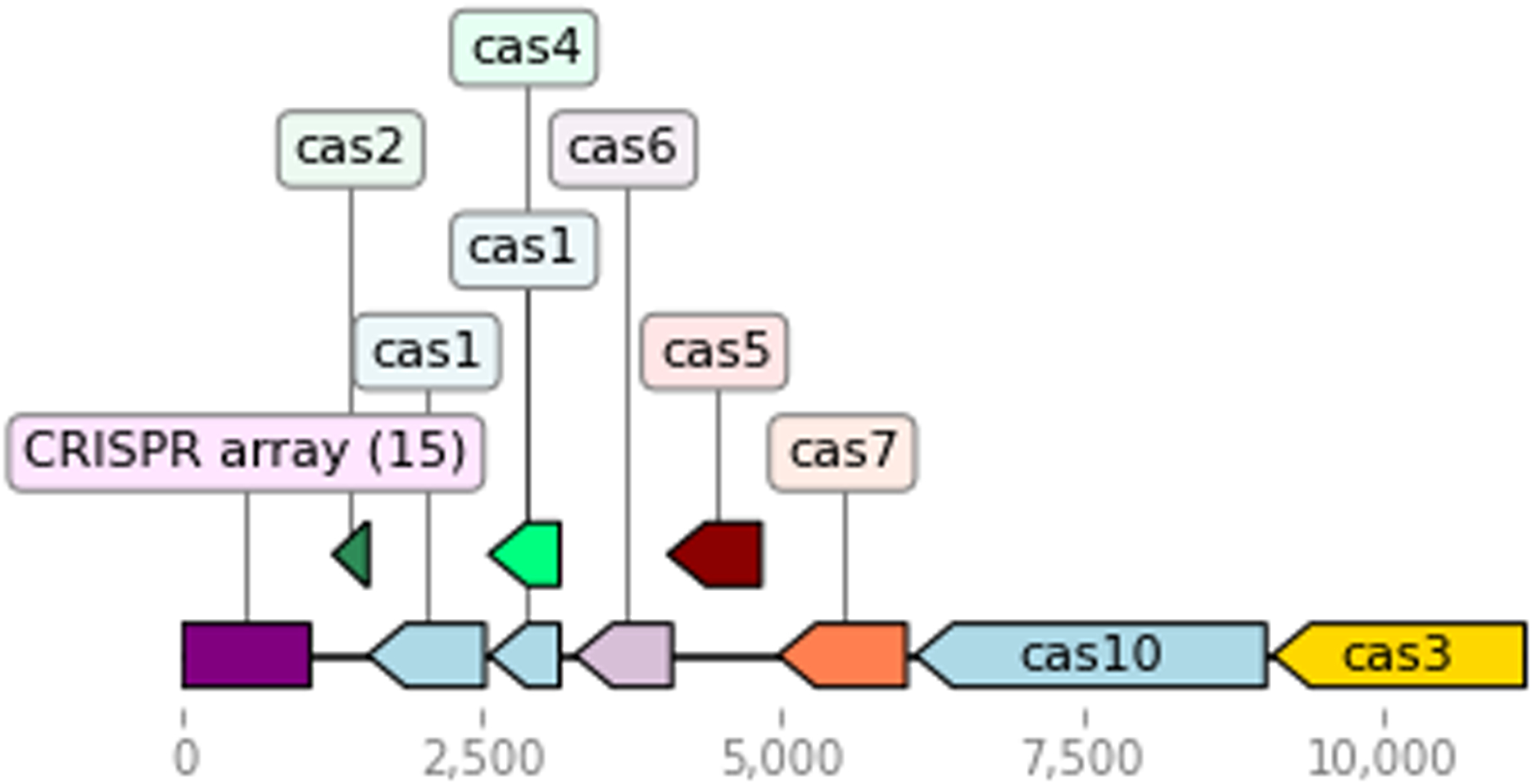
One of two type-I CRISPR-Cas systems present in the genome of *Rippkaea orientalis PCC 8802*. Note that the ORF beginning at position ~2500 has homology with both *cas1* and *cas4*. These alignments have identical bitscores (i.e., the goodness of alignments is quivalent, using this metric), so both annotations appear in the diagram, even though pick_overlapping_features_by_bit_score was applied.

Opfi integrates the DNAFeaturesViewer package (Zulkower & Rosser, 2020) to create gene diagrams of candidate systems. Each input system is visualized as a single PNG image. The sample script below reads in output from the previous example, and generates two gene diagram images, one for each CRISPR-Cas system present in *Rippkaea orientalis*. One image is provided for reference in Figure 1.


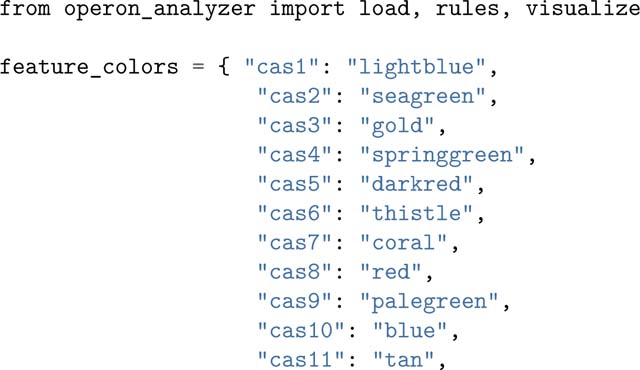



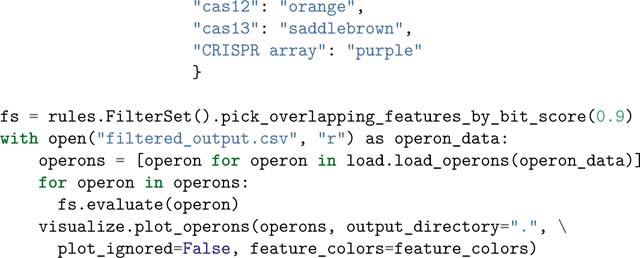


The FilterSet class is used to resolve features with sequences that overlap by more than 90%. Specifically, only the overlapping feature with the highest bitscore value (a quantity that describes the overall quality of an alignment) is rendered when pick_overlapping_fea tures_by_bit_score is applied. Note that is not a requirement for candidate visualization, but can improve gene diagram clarity.
